# No task specialization among helpers in Damaraland mole-rats

**DOI:** 10.1016/j.anbehav.2018.07.004

**Published:** 2018-09

**Authors:** Jack Thorley, Rute Mendonça, Philippe Vullioud, Miquel Torrents-Ticó, Markus Zöttl, David Gaynor, Tim Clutton-Brock

**Affiliations:** a Department of Zoology, University of Cambridge, Cambridge, U.K.; b Kalahari Mole-rat Project, Kuruman River Reserve, Van Zylsrus, South Africa; c Institute of Zoology, University of Neuchatel, Neuchatel, Switzerland; d Department of Zoology and Entomology, Mammal Research Institute, University of Pretoria, Pretoria, South Africa; e Ecology and Evolution in Microbial Model Systems, EEMiS, Department of Biology and Environmental Science, Linnaeus University, Kalmar, Sweden

**Keywords:** Bathyergidae, eusociality, social niche specialization, task allocation, totipotency, trade-offs

## Abstract

The specialization of individuals in specific behavioural tasks is often attributed either to irreversible differences in development, which generate functionally divergent cooperative phenotypes, or to age-related changes in the relative frequency with which individuals perform different cooperative activities; both of which are common in many insect caste systems. However, contrasts in cooperative behaviour can take other forms and, to date, few studies of cooperative behaviour in vertebrates have explored the effects of age, adult phenotype and early development on individual differences in cooperative behaviour in sufficient detail to discriminate between these alternatives. Here, we used multinomial models to quantify the extent of behavioural specialization within nonreproductive Damaraland mole-rats, *Fukomys damarensis*, at different ages. We showed that, although there were large differences between individuals in their contribution to cooperative activities, there was no evidence of individual specialization in cooperative activities that resembled the differences found in insect societies with distinct castes where individual contributions to different activities are negatively related to each other. Instead, individual differences in helping behaviour appeared to be the result of age-related changes in the extent to which individuals committed to all forms of helping. A similar pattern is observed in cooperatively breeding meerkats, *Suricata suricatta*, and there is no unequivocal evidence of caste differentiation in any cooperative vertebrate. The multinomial models we employed offer a powerful heuristic tool to explore task specialization and developmental divergence across social taxa and provide an analytical approach that may be useful in exploring the distribution of different forms of helping behaviour in other cooperative species.

## Highlights

• Cooperative behaviours do not trade off against one another.
• Individuals differ in overall commitment to cooperation.
• Sex differences in behaviour are minimal.
• Cooperative contributions vary with age and relative size.

The morphological and behavioural specialization of individuals to specific tasks is a common feature of complex insect societies (Maynard et al., 1995, Wilson, 1971). To infer specialization it is necessary to show that investment in one cooperative behaviour trades off against investment other forms of cooperative behaviour. In this context, species differ in the extent to which individuals become irreversibly committed to specific roles (Beekman et al., 2006, English et al., 2015), and the extent to which they do so is commonly regarded as an indicator of the complexity of their society on the basis that increased division of labour improves efficiency (Bourke, 1999, Oster and Wilson, 1978; but see Dornhaus, 2008). Some of the most extreme examples are provided by species of eusocial insect where discrete and permanent phenotypic differences exist between functionally sterile workers that focus on different tasks, such as brood care, colony defence or foraging (Bourke and Franks, 1995, Hölldobler and Wilson, 1990, Michener, 1969, Roisin and Korb, 2010). In contrast, in some other social insects, specialization is more labile, and trade-offs are apparent in the form of temporal castes where task allocation varies with age as nonreproductive individuals shift from one role to another; as in honeybees, *Apis mellifera* (Seeley, 1982), some lower termites (Korb and Hartfelder, 2008, Noirot and Pasteels, 1987) and fungus-cultivating ambrosia beetles (Biedermann & Taborsky, 2011). Evidence of behavioural specialization is rare outside of the social insects, but studies of some cooperative mammals have argued that in some species that breed cooperatively, nonreproductive helpers display forms of task specialization analogous to those of castes in social insects.

The case for behavioural specialization in cooperatively breeding mammals has been most strongly advanced for several of the social African mole-rats, including the naked mole-rat, *Heterocephalus glaber*, and the Damaraland mole-rat, *Fukomys damarensis*. In these two species it has been suggested that individuals can be separated into discrete functional groups that differ in their relative contributions to different cooperative activities (Bennett and Faulkes, 2000, Bennett and Jarvis, 1988, Jarvis, 1981, Scantlebury et al., 2006) and their probability of dispersing (O'Riain, Jarvis, & Faulkes, 1996), as well as in related aspects of their size and shape (Bennett & Faulkes, 2000). However, other studies of the distribution of cooperative behaviour in social mole-rats found continuous rather than discrete differences between individuals in their cooperative contributions (Lacey & Sherman, 1991), and a recent study in Damaraland mole-rats has suggested that helpers do not specialize in specific tasks but rather vary in overall helpfulness (Zöttl, Vullioud, et al., 2016).

Determining whether individuals within cooperative societies are behaviourally specialized is more complex than initially appears as the expression of cooperative behaviour can vary between and within individuals in many ways. For example, individuals may differ either in their general contribution to all cooperative activities or in their relative contributions to specific activities. In addition, relative differences in behaviour may be (1) largely driven by age, (2) unrelated to either age or adult phenotype, or (3) associated with contrasts in both adult phenotype and early development, as in the caste systems of many eusocial insects (see Table 1). There may also be many different combinations and subdivisions of the four distributions of cooperative behaviour shown in Table 1. Without longitudinal studies of the behaviour of individuals at different ages, it is often impossible to distinguish between the developmental processes leading to individual differences in behaviour or to allocate societies to different categories. With this information, it is possible to examine the extent to which cooperative behaviours are correlated within individuals, the temporal stability of any correlations across development, and other phenotypic determinants of behaviour, which together underpin the distribution of behaviour across individuals in cooperative societies.

**Table 1:** Forms of individual variation in cooperative behaviour across cooperative societies

Description of variation in cooperative behaviour across individuals	Trade-offs	Early development	Age	Adult phenotype^p^	Examples
Differences in all forms of cooperative behaviour associated with age; temporary and permanent specialization absent	✗	✗	✓	✗	Meerkat, *Suricata suricatta*^a^ White-winged chough, *Corcorax melanorhamphos*^b^ Social spider, *Anelosimus eximius*^c^ Damaraland mole-rat, *Fukomys damarensis*^d^
Specialization in cooperative behaviour independent of age or adult phenotype	✓	✗	✗	✗	Social spider, *Anelosimus studiosus*^e^ Lion, *Panthera leo*^f^ Chimpanzee, *Pan troglodytes*^g^
Specialization in cooperative behaviour associated with age	✓	✗	✓	✗	Princess of Burundi cichlid, *Neolamprologus pulcher*^h^ Honeybee, *Apis mellifera*^i^ Paper wasp, *Polistes canadensis*^j^ Ambrosia beetle, *Xyleborinus saxenseni*^k^
Specialization in cooperative behaviour associated with contrasts in both adult phenotype and early development	✓	✓	✗	✓	Leafcutter ant, *Acromyrmex echinatior*^l^ Big-headed ant, *Pheidole megacephala*^m^ Nasute termite, *Velocitermes barrocoloradensis*^n^ Aphid, *Tuberaphis styraci*^o^

a Clutton-Brock et al. (2003).

b Heinsohn and Cockburn (1994).

c Settepani, Grinsted, Granfeldt, Jensen, and Bilde (2013).

d Zöttl et al. (2016), this study.

e Wright et al. (2014).

f Stander (1992).

g Boesch (2002).

h Bruintjes and Taborsky (2011).

i Seeley (1982).

j Giray, Giovanetti, and West-Eberhard (2005).

k Biedermann and Taborsky (2011).

l Hughes, Sumner, Van Borm, and Boomsma (2003).

m Sameshima, Miura, and Matsumoto (2004).

n Roisin (1996).

o Shibao, Kutsukake, Matsuyama, Fukatsu, and Shimada (2010).

p Qualitative nonbehavioural differences in adult phenotype.

Although earlier studies of social mole-rats have described contrasts in cooperative behaviour between individuals and suggested that they are a consequence of variation in development (Bennett and Jarvis, 1988, Burda, 1990, Lacey and Sherman, 1991), the absence of longitudinal data for individuals has made it impossible to tell whether or not individual differences are a consequence of permanent contrasts in development analogous to those found in insect societies with distinct castes. More recently, Mooney, Filice, Douglas, and Holmes (2015) used a combination of in-group observations and out-of-group tests of pup care and colony defence in naked mole-rats and showed that contributions to different cooperative tasks (work-related tasks, pup care and colony defence) varied across nonbreeding group members in naked mole-rats, and that the expression of these behaviours was stable across time and across litters. They also showed that there was a trade-off between pup care and both colony defence and working behaviour that is suggestive of task specialization. In contrast, recent research on Damaraland mole-rats has shown that individual differences in contributions to cooperative effort are a consequence partly of differences in age and growth and partly of variation in contributions to all forms of cooperative behaviour (including digging, nest building and food carrying: Zöttl, Vullioud, et al., 2016).

Despite these two previous studies using longitudinal data, it is still not fully clear whether or not there is specialization in the relative contributions of individuals to different cooperative activities in either species. In the study of naked mole-rats, specific estimates for individual trade-offs were derived from aggregated observational data collected across a period of days rather than months, and each observation period on groups (30 min) was short in the context of naked mole-rat activity periods (Riccio & Goldman, 2000). In the study of Damaraland mole-rats, behavioural data were similarly aggregated for each individual, and as individuals in the data set were sampled heterogeneously across development, the estimated correlations did not control for variation in age, sex, size or group conditions, all of which are implicated in the expression of cooperative behaviour in other societies (fish: Bruintjes and Taborsky, 2011, Tanaka et al., 2018; mammals: Clutton-Brock, 2016; insects: Field et al., 2006, Thomas and Elgar, 2003; birds: Koenig & Dickinson, 2004). Consequently, it remains unclear what form the distribution of cooperative activity takes in mole-rats and whether or not individuals specialize in particular tasks, as has been suggested (Table 1).

In this paper, we analysed longitudinal records of the development of behaviour in individually marked nonreproductive Damaraland mole-rats to examine individual differences in behaviour and quantify individual correlations across cooperative behaviours to determine whether or not these are negative. We did so using multilevel, multinomial logistic regressions. These statistical models (a form of generalized linear mixed model) are well suited to the structure of observational data but have seldom been used in the context of animal behaviour (see Koster & McElreath, 2017). By treating behaviour as a multinomial response, they overcome the need to aggregate across behavioural categories or across observations within individuals when quantifying individual variation in behaviour (e.g. Arnold et al., 2005, Clutton-Brock et al., 2003, Zöttl et al., 2016), and therefore allow the estimation of individual level variance and within-individual correlations all within the framework of a single model. Trade-offs between different forms of behaviour take the form of negatively correlated random effects, and we therefore used these correlations to elucidate whether mole-rats that regularly engage in one behaviour (e.g. work) also express relatively less of other behaviours (e.g. food carrying).

In addition to asking whether Damaraland mole-rats are behaviourally specialized, we investigated the role of age, group size, relative body mass and sex on cooperative behaviour. We also tested whether the presence of pups affects the expression of care behaviour in nonreproductive mole-rats, through either direct contributions to nest building or increased time spent in the nest. As Damaraland mole-rat pups are highly altricial and hairless, social thermoregulatory benefits derived from huddling might therefore constitute an important form of social and, arguably, cooperative behaviour (Arnold, 1990, Kotze et al., 2008).

## Methods

### Animal Housing and Data Collection

Data were collected from a captive population of Damaraland mole-rats maintained between October 2013 and January 2017 at the Kuruman River Reserve in the Northern Cape of South Africa. All individuals were born in captivity into groups housed in self-contained tunnel systems made of polyvinyl chloride (PVC) pipes modified to have transparent plastic ‘windows’ through which behaviour can be observed. Pipes connected several additional compartments that served as a nestbox, a toilet, a food store and a large waste box. One to three vertical pipes were incorporated into the tunnel design through which clean sand was added. Animals could be recognized individually via a unique coloured dye mark applied to their white head patch, and secondarily via a passive integrated transponder (PIT) tag that was implanted in early life. During observation periods sand was added to the tunnel system at 2 h intervals to increase the expression of ‘work’ behaviours. Animals clear the sand from the vertical pipes and move it through the tunnel system to the peripheral waste box, thereby gaining access to food placed behind the previously sand-filled tunnel. Animals were provisioned twice daily (ad libitum) on a diet of predominantly sweet potatoes and cucumbers. Tunnel systems were cleaned briefly every day and more thoroughly every 2 weeks.

Body mass measurements were acquired by manually removing individuals from their tunnel system and placing them onto an electronic scale. All individuals were weighed approximately every week until the age of 90 days, and every 2 weeks thereafter, yielding mass curves with high resolution. The sex of individuals can be determined from the external genitalia (Seney, Kelly, Goldman, Šumbera, & Forger, 2009).

Behavioural data were derived from instantaneous scan sampling. Intact breeding groups were observed for 12 h in each observation period (hereafter we refer to a single 12 h observation period as a scan), with individual behaviour recorded at 4 min sampling intervals and inputted onto a handheld Android device using the Pocket Observer software (Noldus Information Technology, Wageningen, Netherlands). In this way, 180 sampling events were generated per individual per scan. As our study is concerned with the behaviour of nonreproductive individuals, information from breeding males and females was removed. The analyses were restricted to 10 scan sessions per individual as a compromise between data coverage and computing requirements. The first and last scan were included for all individuals to ensure maximum age coverage, in addition to eight further randomly chosen scans (mean time between scans per individual = 63.82 ± 2.10 days). The total data set considered 60 nonreproductive females and 56 nonreproductive males in 35 groups (mean age at first scan ± = 136.1 ± 0.9 days, mean age at last scan ± = 716.5 ± 14.3 days, mean age span across scans ± = 580.4 ± 13.4 days). The ethogram covers 16 behaviours (Table A1), which were collapsed into six categories for the multinomial modelling: active nonhelping, eating, food carrying, nest building, resting and working. We decided to group all active nonhelping behaviours together so that a distinction could be made between time allocated to helping (food carrying, nest building and working) versus more general patterns of activity.

### Models

The structure of the multilevel, multinomial behaviour models (MMBMs) we employed is outlined in the Appendix. We specified three MMBMs for each sex which differed in the specification of the random effects and the presence or absence of fixed covariates. We analysed the sexes separately so that estimated variance components and behavioural trajectories were sex specific and, by extension, discussions of sex differences in behaviour are qualitatively informed rather than quantitatively informed. We used the widely applicable information criterion (WAIC) to assess the relative fit of models but note that its relevance to the current study is somewhat limited as each model provides uniquely important information about the structuring of behavioural variation in mole-rat societies. WAIC was therefore used as a general indicator rather than a model selection tool, and prominence was instead placed on the model outputs and changes in the apportionment of variance with increasing model complexity.

Model 1 was limited to the intercepts and random effects at the level of the individual, and therefore (1) reveals the extent to which individual level variance is partitioned across behavioural responses and (2) estimates the (within-individual) correlations across these responses. Since individuals were all measured repeatedly for over a year of their life, the within-individual random effects correlations here represent individual behavioural correlations across their development (recall that all individuals were observed an equal number of times). As our study focused on individual trade-offs in time allocation during nonresting periods, resting behaviour was set as the reference category throughout modelling (i.e. correlations between resting and nonresting behaviours were not estimated).

**Figure A1:**
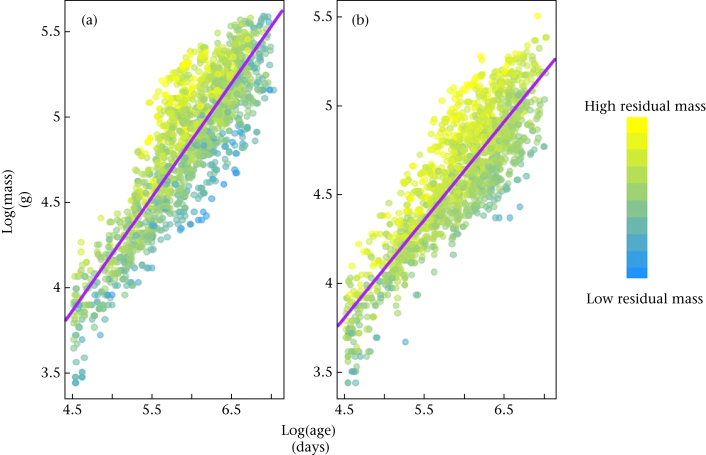
Log-log plots of body mass against age, from which residual mass was extracted, for (a) females and (b) males. The line represents the slope from a simple linear regression, but the residual points for each mass measurement are taken from linear mixed effects models that include a random term for the group; they therefore represent individual mass relative to other same-sex, same-age group mates. To maximize the power of this analysis, all known-aged individuals in the laboratory population were used, regardless of whether they had enough behavioural information to be included in the multinomial behavioural models.

Model 2 retained the random effects at the level of the individual and incorporated several fixed covariates that were hypothesized to be important ecological predictors of behaviour in mole-rats. Since Model 2 differed from Model 1 only in the specification of fixed effects, comparing these first two models provides some information about how much individual level variance in behavioural categories is accounted for by the fixed effects (notwithstanding some caveats: Koster & McElreath, 2017). Because the expression of behaviour in cooperative breeders is often age dependent, age was included as a first-, second- and third-order polynomial (Zöttl, Vullioud et al., 2016). It was anticipated that group level processes could also mediate behavioural time budgets and contributions to cooperation, so group size was specified as a first- and second-order polynomial. A categorical covariate for the presence of pups was included to test our hypothesis that individuals spend more time in the nest when pups are present; we classed pups as animals less than 40 days old. Lastly, as it is common in cooperative societies for individual state to influence contributions to cooperative behaviour, a term for ‘relative mass’ was included that used the body mass of the animal recorded closest to the observation period. In order that mass was estimated relative to other same-sex, same-age group members, ‘relative mass’ represented the residuals from sex-specific linear mixed models that fitted log(mass) as a function of log(age), in the presence of a random term for group identity (Fig. A1). All continuous covariates were *z* score transformed before model fitting.

Model 3 retained the structure of Model 2 and included further random effects at the level of the scan, the litter and the group. The inclusion of scan level random effects controlled for the temporal pseudoreplication introduced by using repeated observations from individuals within a single scan. There was also clustering in the data at the level of the group and the litter; the number of observations at each of these levels was modest, which presumably places low confidence around the estimation of their variances. Their inclusion should none the less refine the estimation of the fixed effects. The addition of further random effects also changes the interpretation of the individual level variances and the within-individual random effects correlations. Notably, the individual level random effects no longer represent the deviations from the population level average, making this model unsuited to the estimation of individual trade-offs. Instead, Model 3 was used to describe general effects on the distribution of cooperative behaviour between the sexes and across individuals.

Models were fitted and assessed using the RStan and rethinking packages in R, respectively, under a Bayesian framework. In comparison to traditional Markov chain Monte Carlo approaches, RStan makes use of a Hamiltonian Monte Carlo algorithm for model estimation that requires many-fold fewer iterations before posterior distributions are mixed. We specified three chains of 1000 iterations for every model, half of which were allocated to the warm up. As per Koster and McElreath (2017), a noncentred parameterization of the random effects was specified, using a Cholesky factorization of the variance - covariance matrices. Weakly informative priors were set for the fixed effects parameters and the variance–covariance matrices and were chosen so that the data influenced the posterior values as much as possible (i.e. priors had only a weak influence on the posterior distribution). Model diagnostics highlighted sufficient mixing of chains for all models.

The significance of the correlated random effects in Models 1 and 2 was evaluated from the credible intervals of their posterior distributions, such that a biologically important effect was inferred in cases where the 95% credible intervals did not overlap zero. For the continuous fixed effects in Models 2 and 3, the predicted probabilities were emphasized above the raw model coefficients for the posterior means, as the latter were difficult to interpret directly because of their relationship to the reference category. The predicted probabilities were only calculated from the fixed effects. For the single categorical fixed effect (presence of pups), we followed the advice of Koster and McElreath (2017) and used the distribution of the contrasts from each posterior sample to test significance, rather than prediction intervals; the intervals incorporate uncertainty from the other fixed covariates and therefore reduce the confidence with which differences between categorical factors can be assessed. We provide the R code for our analyses as Supplementary Material.

### Ethical Note

All the research carried out in this study was approved by the University of Pretoria animal ethics committee (permit numbers EC089-12 and SOP-004-13). All data collection was observational and therefore unlikely to have caused any harm to animals. The implantation of transponders in early life was carried out under anaesthesia when animals were larger than 40 g (Identipet ISO FDX-B Microchip, 12 mm by 2.1 mm, 0.06 g; Identipet (Pty) Ltd., Johannesburg, South Africa).

## Results

In presenting the results, we first present the evidence for within-individual trade-offs in behaviour, before dealing with general effects on the distribution of cooperative behaviour between the sexes and across individuals. As expected, WAIC comparisons highlighted a successively better fit with increasing model complexity (Table A2), so the presentation of general effects of age, relative body mass and group size is restricted to Model 3 for each sex.

### Within-Individual Trade-Offs

**Figure 1:**
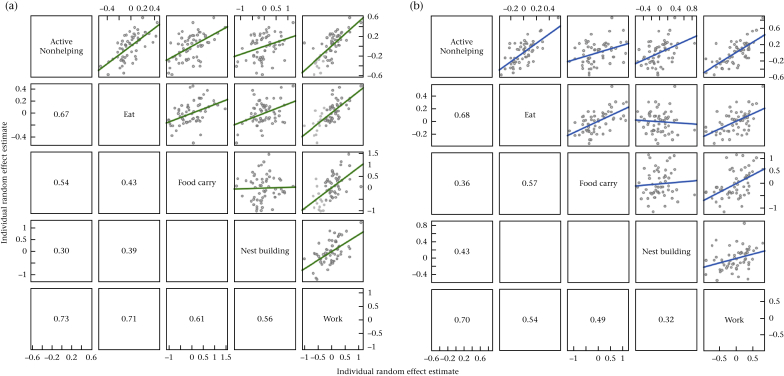
Within-individual random effects correlations from Model 2, for (a) females and (b) males. Note that the values presented in the lower half of the matrix represent the correlations between the median individual level intercept in the posterior samples for each behaviour; they are therefore larger than the correlations presented in Table 2, which are taken directly from the variance–covariance matrices of the posterior samples.

Our analyses provided no evidence for task specialization. The presence of task specialization is predicated on negative correlations between different cooperative behaviours within individuals (trade-offs), but in no case did we detect a significant negative correlation between two behaviours (excluding the reference category of resting). Instead, nonresting behaviours were positively correlated across development (Table A3, Table A4, for random effects correlations from all models in females and males, respectively), which suggests that individuals that frequently exhibit one nonresting behaviour also tend to have a high probability of engaging in other nonresting behaviours (Table 2). This trend extends to cooperative behaviours: females that worked relatively more across their development than the population average were also more frequently observed nest building (ρ_4,5_ = 0.31 ± 0.12) and food carrying (ρ_3,5_ = 0.16 ± 0.11), and males that were more frequently observed working also engaged more often in food carrying (ρ_4,8_ = 0.24 ± 0.11). Most of the correlations were strengthened by the addition of fixed effects (Table 2, lower half of each matrix), so that after having controlled for general factors affecting behaviour, positive associations between cooperative behaviours predominated (Fig. 1 from Model 2).

**Table 2:** Correlations of random effects across the responses in each of the tested models

	Active nonhelping	Eat	Food carry	Nest building	Work
Females					
Active nonhelping		**0.49 (0.12)**	**0.34 (0.12)**	0.28 (0.14)	**0.60 (0.09)**
Eat	**0.54 (0.11)**		0.10 (0.15)	0.07 (0.17)	0.14 (0.12)
Food carry	**0.43 (0.12)**	0.07 (0.15)		−0.13 (0.15)	**0.16 (0.11)**
Nest building	0.19 (0.14)	0.17 (0.17)	−0.13 (0.15)		**0.31 (0.12)**
Work	**0.63 (0.08)**	**0.28 (0.12)**	**0.22 (0.10)**	**0.30 (0.11)**	
Males					
Active nonhelping		**0.62 (0.10)**	**0.30 (0.12)**	0.30 (0.17)	**0.68 (0.08)**
Eat	**0.55 (0.11)**		0.19 (0.16)	−0.31 (0.21)	0.11 (0.12)
Food carry	0.24 (0.13)	**0.35 (0.15)**		−0.04 (0.20)	**0.24 (0.11)**
Nest building	0.27 (0.16)	−0.31 (0.20)	0.08 (0.19)		0.00 (0.16)
Work	**0.61 (0.09)**	0.08 (0.13)	**0.22 (0.12)**	0.02 (0.16)	

The upper half of each matrix denotes correlations from Model 1 for each sex, the lower half correlations from Model 2. Estimates represent the means from the posterior samples (SD in parentheses). Parameters in bold indicate estimates where the 95% credible intervals do not span zero.

### General Effects on Mole-rat Behaviour

Sex differences in overall time budgets were minimal, with males and females allocating similar amounts of time to each behaviour (coefficients of intercepts, Table A5). The individual variances associated with the behaviours also showed parity between the sexes (Table A6 contains all random effects variances). Behaviours with low variance characterize activities that were distributed relatively evenly across individuals, such as eating and active nonhelping behaviour, while some of the less common activities (nest building and food carrying) display high variances and were therefore less consistently expressed across individuals. Since work behaviour was expressed often in males and females but displays a relatively modest individual level variance, this suggests that all individuals engage in appreciable levels of work behaviour.

**Figure 2:**
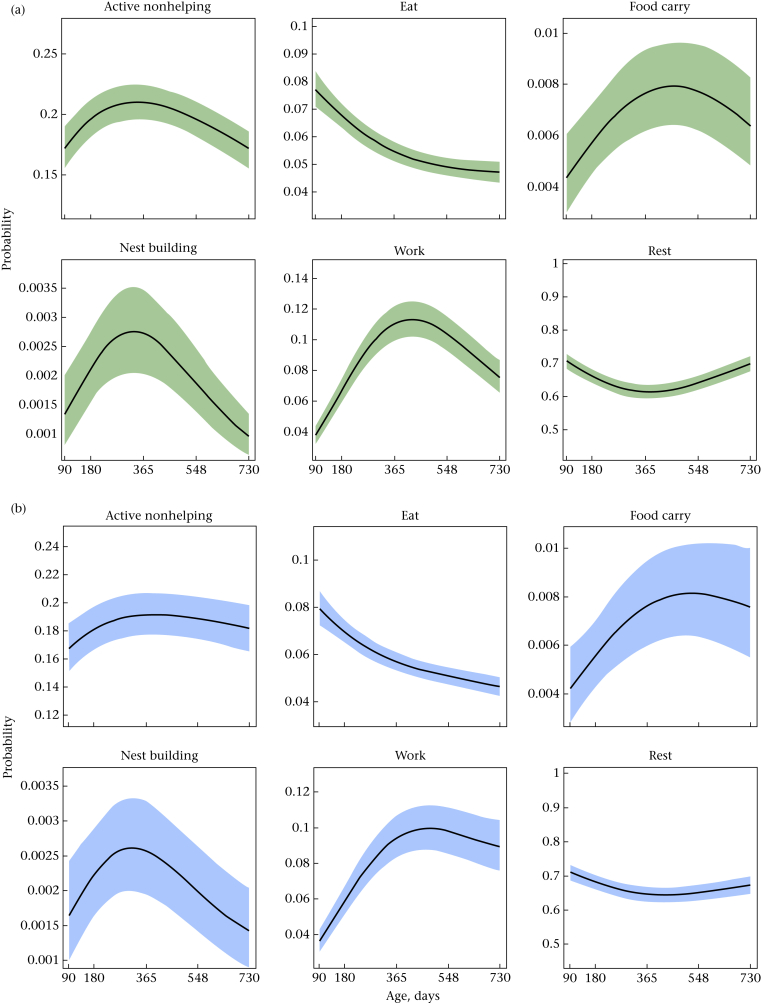
Model predictions of response behaviours with changing age, for (a) females and (b) males. All other fixed covariates are held at sample mean, with shaded regions specifying the 89% percentile intervals, calculated from the posterior samples of Model 3 for each sex.

**Figure 3:**
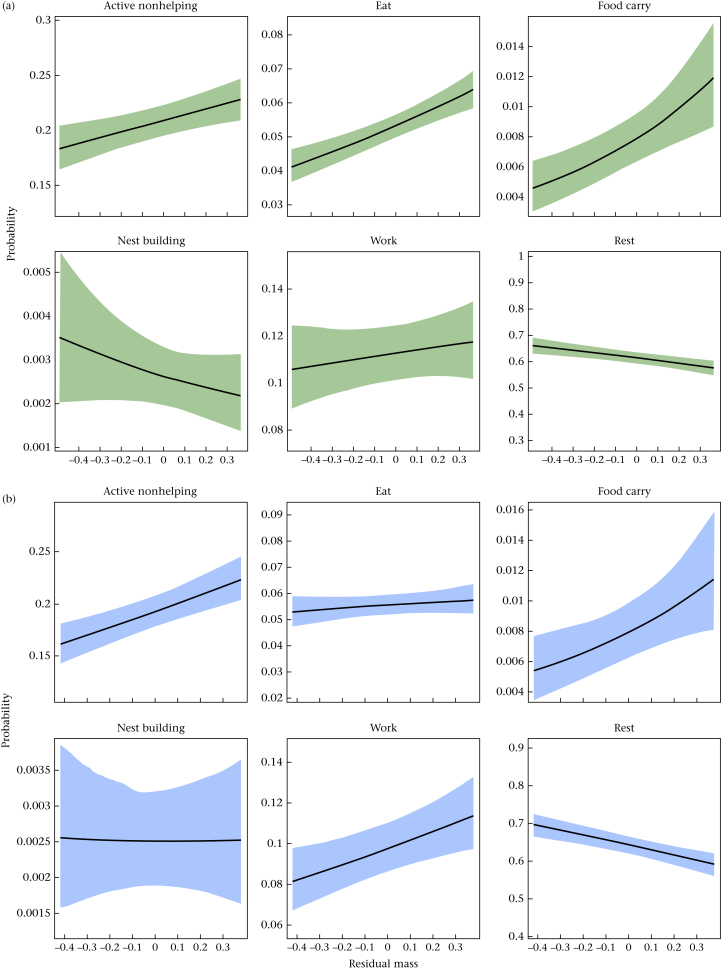
Model predictions of response behaviours with changing relative size, for (a) females and (b) males. All other fixed covariates are held at the sample mean, with shaded regions specifying the 89% percentile intervals, calculated from the posterior samples of Model 3 for each sex.

Age and relative body mass were both major determinants of cooperative contributions in these Damaraland mole-rats (Figure 2, Figure 3, Table A7). With respect to age, most behaviours displayed nonlinear patterns (Fig. 2). Total activity is reflected in the inverse of the predicted curve for rest, indicating that total activity increased until 1 year of age, before declining. This general trend in activity was mirrored by analogous age-related changes in cooperative behaviour, with nest-building, food-carrying and work behaviours all being expressed increasingly frequently in the first year of life. Nest-building behaviour peaked particularly early, at around 9 months and showed steep declines after this point. The degree to which time allocated to work declined in midlife seems to be sex dependent, as marked declines in this behaviour were only apparent in females. With respect to relative body mass, increases in body mass were associated with reductions in nest building in females (invariant in males), but after fixing age to the mean value across the data set (400 days for females, 396 days for males), a larger relative body mass was associated with increases in both food-carrying and work behaviour (Fig. 3), the latter effect being stronger in males.

**Figure 4:**
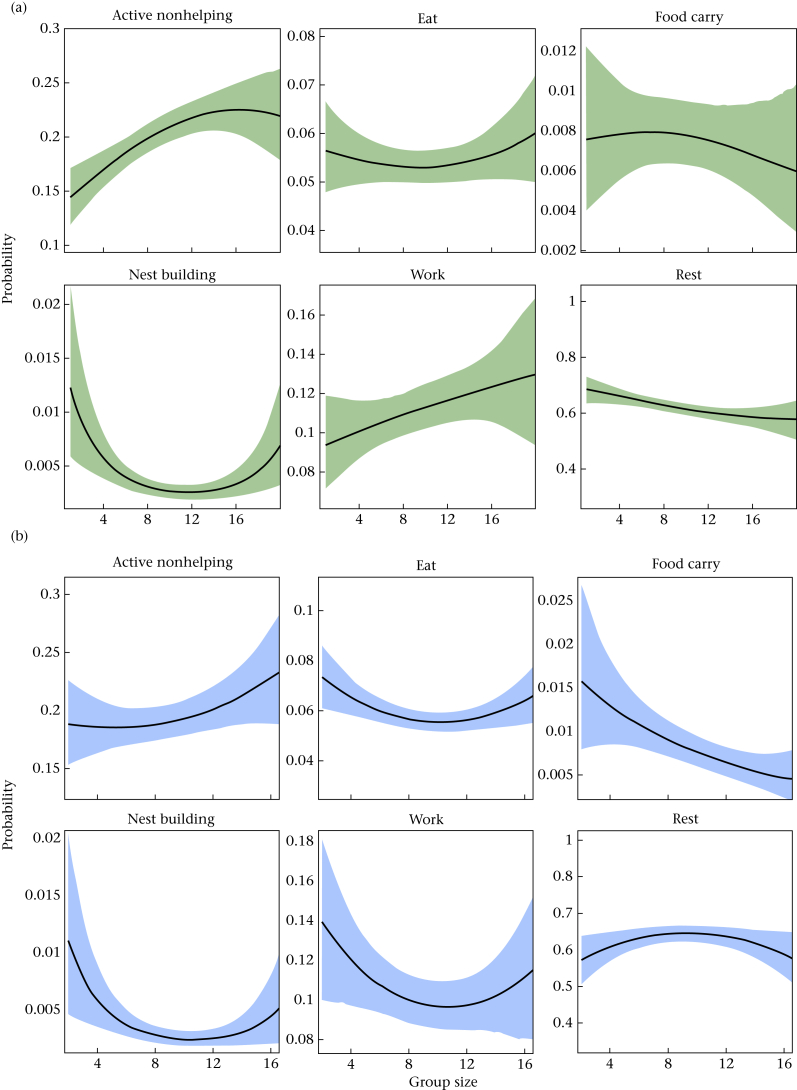
Model predictions of response behaviours with changing group size, for (a) females and (b) males. All other fixed covariates are held at the sample mean, with shaded regions specifying the 89% percentile intervals, calculated from the posterior samples of Model 3 for each sex.

Individual behaviour was also influenced by group size (Fig. 4) and, in most cases, the visualization of quadratic trends suggests that these effects manifest themselves at the upper and lower boundaries of group sizes, where confidence surrounding the estimates is weaker. Nevertheless, the models suggest that the effect of group size on work is sex dependent (Fig. 4, Table A7), with increases in group size raising workload in females and reducing workload in males in a quadratic fashion. Beyond this, several behaviours displayed linear relationships with group size, most notable being the reduction in resting behaviour and food-carrying behaviour in females and males, respectively.

**Figure A2:**
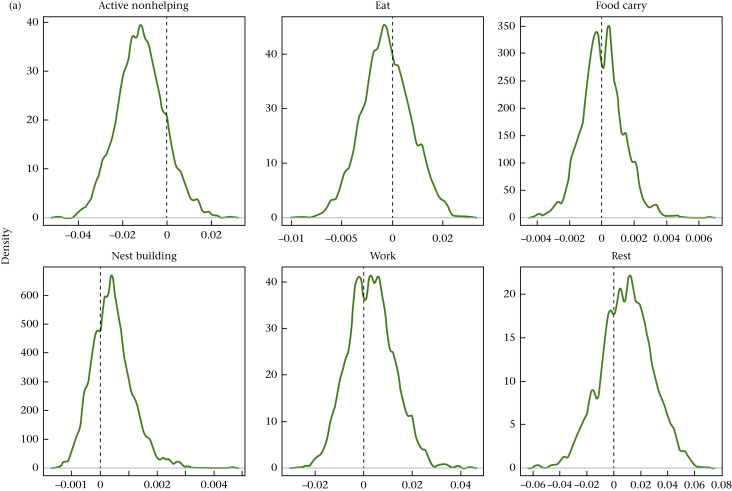

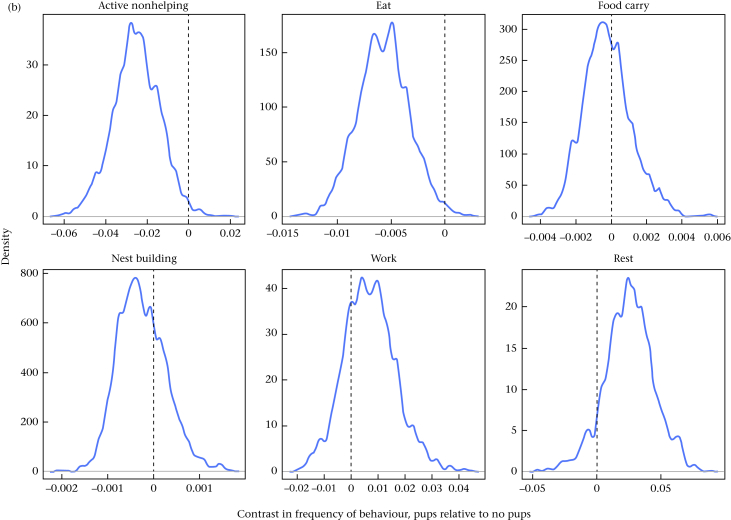
Distributions of posterior contrasts for pup presence; the contrast from each sample in the posterior, for (a) females and (b) males. This method is preferred over the prediction intervals as per the continuous covariates, as the latter incorporate uncertainty from all the parameters in Model 3 and therefore offer less confidence in assessing differences between categorical covariates.

Males and females did not spend more time in the nest when pups were present (Fig. A2), and other aspects of cooperative behaviour were similarly unaffected by the presence of pups (Table A7).

## Discussion

Our analysis found no evidence for task specialization in nonreproductive Damaraland mole-rats. If present, task specialization should be detectable in the form of within-individual trade-offs between functionally divergent behaviours. Instead, we found that individual correlations across nonresting behaviours were consistently positive, indicating that individual mole-rats that are more active and spend more time away from their nest tend to engage more in all forms of cooperative behaviour: food carrying, nest building and work behaviour.

Any division of labour over workload that has previously been suggested in Damaraland mole-rats from direct observations in captivity (Bennett, 1990, Bennett and Jarvis, 1988) or indirect measures of activity in the wild (Scantlebury et al., 2006) probably stems from variation in the cooperative contributions of cohorts of animals at different developmental stages and thus sizes (see also Zöttl, Thorley, Gaynor, Bennett, & Clutton-Brock, 2016), each of which will affect the relative energetic costs of helping (Clutton-Brock, 2016, McNamara and Houston, 1996). The absence of longitudinal sampling from known-aged individuals in earlier studies made it impossible to determine whether the cooperative contributions of individuals were due to age or to divergent developmental trajectories in the sense of permanent castes. By incorporating information from known-aged individuals, it has become clear that age is a key determinant of cooperative behaviour (Zöttl, Vullioud, et al., 2016; this study) and the case for permanent castes has been refuted on the basis that all cooperative behaviours show the same trajectory: increasing during ontogeny and decreasing after reaching asymptotic mass. However, it remained possible that individuals could none the less be specialized in their cooperative contributions as they age in a manner that might mirror the temporal castes of honeybees (Seeley, 1982), doing qualitatively more or less of different cooperative activities as they age. Here, in failing to find any evidence of specialization (trade-offs) across cooperative behaviours in this study, we also refute the case for temporal castes and, consequently, it seems that the behavioural differentiation of individuals in Damaraland mole-rat groups is fundamentally different to that observed in eusocial insects, where labour divisions among nonreproductives are associated with behavioural and/or morphological specialization (Boomsma & Gawne, 2018).

Our results add further information regarding the factors affecting behavioural expression in Damaraland mole-rats. We found that the ontogenetic trajectories of behaviour of nonreproductive males and females were extremely similar in both shape and magnitude. Nest-building behaviour peaked particularly early, at around 9 months, and showed steep declines in individuals after this point. This relatively infrequent behaviour was therefore mostly performed by young nonbreeders of both sexes, perhaps reflecting the lower energetic requirements of nest building compared to working and food-carrying behaviour (see Zöttl et al., 2018, where the same argument has been put forward for pup-carrying behaviour). The lack of overall sex differences deviates somewhat from other cooperative breeders where a substantial component of variation in behaviour is due to sex (Clutton-Brock, 2016, Clutton-Brock et al., 2002, Hodge, 2007) and might reflect the similarly negligible opportunities for independent breeding in subordinates of each sex in Damaraland mole-rats, which would be expected to minimize sex-specific divergence in helping strategies (Holmes, Goldman, Goldman, Seney, & Forger, 2009). The extreme reproductive suppression of subordinate females (Molteno & Bennett, 2000) presumably also prevents the evolution of allolactation in the social mole-rats, with females effectively entering a state of suspended development until reproduction stimulates a secondary burst of ‘puberty-like’ growth and the onset of sexual characteristics (Dengler-Crish & Catania, 2007; Thorley, Katlein, Goddard, Zöttl, & Clutton-Brock, 2018). One exception where sex differences in helping are apparent in mole-rats is pup care in the form of pup carrying, which has previously been shown to be more frequently performed by females (Zöttl et al., 2016, Zöttl et al., 2016). We could not investigate this association in this study because we excluded pup carrying from our analysis as it was extremely rarely observed. This decision was based on statistical grounds as rare behaviours are not well accommodated in our modelling framework (Koster & McElreath, 2017); incorporating additional information from standardized behavioural assays could be particularly informative when this occurs (e.g. Mooney et al., 2015).

Sex differences aside, the distribution of cooperative behaviour among individuals in Damaraland mole-rats resembles that in meerkats, *Suricata suricatta*. Meerkats show a more diverse array of cooperative behaviours than mole-rats, including allolactation, babysitting and pup feeding as well as burrow digging and group defence. Males contribute more to sentinel duty than females, which contribute more to babysitting and pup feeding (Clutton-Brock et al., 2002) but, as in Damaraland mole-rats, all meerkat helpers engage in the full range of activities, and show no evidence of individual specialization in specific forms of cooperation (Clutton-Brock et al., 2003): relatively heavy female helpers contribute more to most cooperative activities in their first year of life, and in adulthood cooperative contributions are instead largely driven by increases in daily weight gain, an index of foraging success (Clutton-Brock et al., 2001). The general commitment of different individuals to all forms of cooperative behaviour increases up to the second year of life and shows a tendency to decline in older helpers, which disperse shortly after (Clutton-Brock et al., 2003). Similar processes are likely to explain the age-related declines in helping seen in mole-rats. The precise timing of dispersal in mole-rats in the wild is hard to determine because of the difficulties of ageing wild mole-rats. Nevertheless, loss of individuals from intact groups and recaptures of dispersive individuals suggest that individuals of both sexes remain philopatric for 12–18 months before dispersing (Torrents-Ticó, Bennett, Jarvis, & Zöttl, n.d.). This timing matches the declines in helping behaviour seen in captivity. However, if anything, the declines in helping behaviour are more prominent in females, which is at odds with the evidence that males disperse slightly earlier, and more frequently, than females (Hazell et al., 2000, Torrents-Ticó et al.,).

That two species of cooperatively breeding mammal fail to show evidence of task specialization raises important questions about its presence in naked mole-rats. Naked mole-rats remain one of the strongest candidates for task specialization in the vertebrates, displaying high reproductive skew, extreme group sizes (up to 295 individuals: Brett, 1991, Jarvis and Bennett, 1993) and socially induced infertility in nonbreeders (Faulkes et al., 1990, Faulkes et al., 1991), which together would be expected to increase selection for a nonreproductive division of labour with task allocation (Bourke, 1999). As we have described, Mooney et al. (2015) suggested that task specialization occurs in nonreproductive naked mole-rats, based on evidence of individual consistency in relative contributions to different cooperative activities. However, although they showed that contributions to pup care are negatively related to work (digging and colony maintenance) and defensive behaviour, these trade-offs are founded upon observational data collected over a period of days rather than the period of months that was used in the same study to demonstrate behavioural consistency within individuals. Their inference of specialization is therefore indirect and relies on the combined presence of short-term trade-offs and longer-term consistency. At no point were trade-offs measured throughout the development of individuals, as is necessary when testing for long-term specialization. In addition, the ages of individuals included in their analyses are not clear. We believe that, as yet, it is uncertain whether specialization and caste formation occur in nonreproductive naked mole-rats and that further longitudinal data are necessary (i.e. to ascertain where they should fit in Table 1).

Firm evidence of task specialization in other nonhuman social vertebrates is also scarce. Some of the most frequently cited examples of specialization refer to societies engaged in coordinated hunts where individuals repeatedly adopt specific roles, as has been reported in African lions, *Panthera leo* (Stander, 1992), bottlenose dolphins, *Tursiops truncatus* (Gazda, Connor, Edgar, Cox, & Bar, 2005), and chimpanzees, *Pan troglodytes* (Boesch, 2002). In the case of bottlenose dolphins in Florida, ‘drivers’ consistently herded fish towards other barrier-forming group members, corralling them into tight shoals, improving the hunting efficiency of the group. In African lionesses, increases in hunting success were achieved by females repeatedly adopting either a peripheral stalking role or a central attacking role. Presumably such coordinated hunting relies on relatively stable groups where individuals recognize one another and interact repeatedly, allowing individuals to practise and perfect the specific motor controls for their role within what could be defined as a ‘team’ (Anderson & Franks, 2001; albeit many social animals do not have such defined roles when hunting in groups: Lang & Farine, 2017). Other putative examples of specialization have been presented outside the context of group hunting, and these cases refer more strictly to individual level trade-offs across cooperative tasks. In cooperatively breeding noisy miners, *Manorina melanocephala*, Arnold et al. (2005) found a negative correlation between helper investment in chick provisioning and predator defence that is indicative of specialization if maintained across multiple breeding attempts, and in the mound-building mouse, *Mus spicilegus*, task-related consistency was apparent when collective mound building was induced in captivity (Hurtado, Fénéron, & Gouat, 2013). These aside, other cases are limited. This might in part reflect research effort, as few studies appear to have set out with the aim of testing for individual trade-offs within or across cooperative behaviours throughout development. However, given that its quantification falls into the wider and highly topical agenda in behavioural ecology to quantify individual variation in behaviour (often in the context of ‘animal personality’, ‘behavioural syndromes’, or ‘social niche specialization’: Bergmüller et al., 2010, Jandt et al., 2014, Montiglio et al., 2013, Walton and Toth, 2016, Wright et al., 2014), it seems probable that task specialization involving trade-offs across cooperative tasks is uncommon outside of the insects, and that where specialization does occur in vertebrates it will more often involve cognitively demanding tasks requiring multiple individuals to cooperate in teams towards a single goal, rather than largely individual tasks where group members receive benefits indirectly.

Studies of the structure of animal societies commonly need to ask questions about the extent and distribution of individual differences in behaviour. Do individuals follow different social trajectories? Do they specialize in certain roles across development? Are specializations transient, sequential or irreversible? Are contrasts in development related to changes in gene function or in genotype? We believe that the multinomial models we employed are well suited to address questions of this kind in many different taxa and can provide a common quantitative framework which will make it possible to discriminate the different ways in which individual differences in behaviour develop.

## Author contributions

J.T. conceived the study and performed the statistical analyses. All authors contributed to the organization of the research site where the data were collected; R.M., P.V., M.T. and M.Z. organized and carried out much of the data collection for this study. J.T. and T.C-B. wrote the paper, with input from the other authors at various stages. All authors gave final approval for publication.

## Conflict of interest statement

The authors declare no conflict of interest in the study.

## Data availability

Data have been deposited in the University of Cambridge Data Repository https://doi/org/10.17863/CAM.23196.

## Figures and Tables

**Table A1:** Damaraland mole-rat ethogram

Response category^a^	Subcategories	Description of behaviour
Active nonhelping behaviour	Gnaw	Gnawing on the tunnel walls with teeth
	Locomotion	Moving through the tunnel system, but not engaged in obvious work
	Pump	Characteristic, repetitive up and down movement of the rear body part of the individual
	Other	Behaviours not assignable to the above categories
	Self-groom	Self-directed grooming
	Sniff	Investigating something with the nose
	Social interaction	Sparring with incisors, tail pulling, biting, copulation, dominance interaction
Eat	Eat	Eating food
Food carry (helping)	Food carry	Transporting food either by pushing it along the tunnels (forward) or by dragging it while moving backwards
Nest building (helping)	Nesting material	Individual engaged with paper, either dragging in the direction of the nest, chewing it into small pieces or trying to pull it out of a certain location
Rest	Huddling	Resting in body contact with other individuals in the tunnel (in sight)
	Rest	Individuals resting in the nest (out of sight)
Work (helping)	Dig	Using extrabuccal teeth and front paws to dig in the sand or blockage of paper
	Kick	Pushing sand into tunnel gaps or other locations with the hindlegs or with the nose. Often used to block feeders or tunnel gaps
	Sweep	Sweeping sand with hindpaws through the tunnel system, often to the waste box
	Locomotion between work	Moving between episodes of the above behaviours

a Pup carrying (the retrieval of pups that have left the nest) was excluded from the ethogram as it was observed very few times across all the scans in the data set.

**Table A2:** Model comparisons for the four models fitted to female and male data sets

	Model	Random effects	Fixed effects	WAIC (SE)	ΔWAIC	Mass
Female						
	1	Individual	N	242 504.2 (636.83)	7466.6	0
	2	Individual	Y	241 569.3 (635.96)	6531.7	0
	3	Individual, scan, group, litter	Y	235 037.7 (637.42)	0	1
Male						
	1	Individual	N	222 472.9 (613.72)	6529.6	0
	2	Individual	Y	221 382.8 (612.78)	5439.5	0
	3	Individual, scan, group, litter	Y	215 943.3 (615.10)	0	1

WAIC: widely applicable information criterion.

**Table A3:** Correlations of random effects across the behavioural responses in each of the tested models in females

Sex	Model/random effect	Behaviour	Behaviour				
			Active nonhelping	Eat	Food carry	Nest building	Work
Female	1, individual level	Active nonhelping		0.49 (0.12)	**0.34 (0.12)**	0.28 (0.14)	**0.60 (0.09)**
		Eat	–		0.10 (0.15)	0.07 (0.17)	0.14 (0.12)
		Food carry	–	–		−0.13 (0.15)	0.16 (0.11)
		Nest building	–	–	–		**0.31 (0.12)**
		Work	–	–	–	–	
Female	2, individual level	Active nonhelping		**0.54 (0.11)**	**0.43 (0.12)**	0.19 (0.14)	**0.63 (0.08)**
		Eat	–		0.07 (0.15)	0.17 (0.17)	**0.28 (0.12)**
		Food carry	–	–		−0.14 (0.15)	**0.22 (0.30)**
		Nest building	–	–	–		**0.58 (0.33)**
		Work	–	–	–	–	
Female	3, individual level	Active nonhelping		**0.76 (0.12)**	**0.44 (0.17)**	**0.46 (0.20)**	**0.64 (0.10)**
		Eat	–		0.04 (0.28)	0.22 (0.29)	0.12 (0.23)
		Food carry	–	–		−0.21 (0.25)	**0.25 (0.17)**
		Nest building	–	–	–		0.29 (0.22)
		Work	–	–	–	–	
Female	3, scan level	Active nonhelping		**0.86 (0.03)**	**0.66 (0.05)**	**0.38 (0.07)**	**0.84 (0.02)**
		Eat	–		**0.20 (0.10)**	0.07 (0.13)	0.11 (0.07)
		Food carry	–	–		0.08 (0.11)	**0.13 (0.05)**
		Nest building	–	–	–		**0.09 (0.05)**
		Work	–	–	–	–	
Female	3, group level	Active nonhelping		−0.02 (0.35)	0.09 (0.34)	−0.04 (0.34)	0.04 (0.36)
		Eat	–		−0.08 (0.33)	0.04 (0.36)	0.05 (0.34)
		Food carry	–	–		−0.01 (0.36)	0.01 (0.36)
		Nest building	–	–	–		0.03 (0.35)
		Work	–	–	–	–	
Female	3, litter level	Active nonhelping		0.02 (0.35)	0.05 (0.36)	−0.00 (0.35)	0.05 (0.35)
		Eat	–		0.05 (0.35)	0.06 (0.34)	0.02 (0.34)
		Food carry	–	–		0.01 (0.35)	0.10 (0.35)
		Nest building	–	–	–		0.05 (0.36)
		Work	–	–	–	–	

Estimates represent the means from the posterior samples (SD in parentheses). Parameters in bold indicate estimates where the 95% credible intervals do not span zero.

**Table A4:** Correlations of random effects across the behavioural responses in each of the tested models in males

Sex	Model/random effect	Behaviour	Behaviour				
			Active nonhelping	Eat	Food carry	Nest building	Work
Male	1, individual level	Active nonhelping		**0.62 (0.10)**	**0.30 (0.12)**	0.30 (0.17)	**0.68 (0.08)**
		Eat	–		0.19 (0.16)	−0.31 (0.21)	0.11 (0.12)
		Food carry	–	–		−0.04 (0.20)	**0.24 (0.11)**
		Nest building	–	–	–		−0.01 (0.16)
		Work	–	–	–	–	
Male	2, individual level	Active nonhelping		**0.55 (0.11)**	0.24 (0.13)	0.27 (0.16)	**0.61 (0.09)**
		Eat	–		**0.35 (0.15)**	−0.31 (0.20)	0.08 (0.13)
		Food carry	–	–		0.08 (0.19)	**0.22 (0.12)**
		Nest building	–	–	–		0.02 (0.16)
		Work	–	–	–	–	
Male	3, individual level	Active nonhelping		**0.63 (0.16)**	0.32 (0.26)	0.07 (0.33)	**0.66 (0.15)**
		Eat	–		0.24 (0.26)	−0.26 (0.33)	0.25 (0.21)
		Food carry	–	–		0.11 (0.33)	0.26 (0.19)
		Nest building	–	–	–		−0.04 (0.24)
		Work	–	–	–	–	
Male	3, scan level	Active nonhelping		**0.83 (0.04)**	**0.51 (0.06)**	**0.42 (0.09)**	**0.80 (0.03)**
		Eat	–		**0.39 (0.12)**	0.18 (0.16)	0.14 (0.08)
		Food carry	–	–		−0.08 (0.15)	0.10 (0.08)
		Nest building	–	–	–		−0.10 (0.08)
		Work	–	–	–	–	
Male	3, group level	Active nonhelping		0.09 (0.34)	**−0.02 (0.34)**	0.04 (0.36)	0.18 (0.36)
		Eat	–		0.04 (0.35)	0.07 (0.34)	−0.16 (0.33)
		Food carry	–	–		−0.03 (0.35)	0.04 (0.35)
		Nest building	–	–	–		0.07 (0.33)
		Work	–	–	–	–	
Male	3, litter level	Active nonhelping		0.24 (0.36)	0.26 (0.33)	0.24 (0.33)	0.45 (0.36)
		Eat	–		0.22 (0.30)	−0.13 (0.33)	−0.06 (0.29)
		Food carry	–	–		0.11 (0.30)	0.16 (0.28)
		Nest building	–	–	–		0.11 (0.29)
		Work	–	–	–	–	

Estimates represent the means from the posterior samples (SD in parentheses). Parameters in bold indicate estimates where the 95% credible intervals do not span zero.

**Table A5:** Posterior means (SD in parentheses) of the intercepts in each model, i.e. expression of behaviour relative to resting

	Active nonhelping	Eat	Food carry	Nest building	Work
Model 1, female	−1.22 (0.03)	−2.43 (0.03)	−4.43 (0.09)	−5.12 (0.09)	−1.94 (0.05)
Model 2, female	−1.15 (0.04)	−2.49 (0.04)	−4.34 (0.10)	−5.20 (0.12)	−1.75 (0.05)
Model 3, female	−1.08 (0.06)	−2.45 (0.05)	−4.37 (0.14)	−5.47 (0.16)	−1.70 (0.08)
Model 1, male	−1.25 (0.04)	−2.42 (0.03)	−4.34 (0.10)	−5.36 (0.08)	−1.96 (0.06)
Model 2, male	−1.18 (0.04)	−2.46 (0.04)	−4.22 (0.11)	−5.34 (0.11)	−1.79 (0.06)
Model 3, male	−1.21 (0.07)	−2.45 (0.05)	−4.42 (0.15)	−5.56 (0.17)	−1.90 (0.09)

**Table A6:** Variance estimates of the random effects in the six models tested in the paper

Random effect	Female			Male		
	Model 1	Model 2	Model 3	Model 1	Model 2	Model 3
**Individual level**						
Active nonhelping	0.43 (0.63)	0.27 (0.03)	0.20 (0.02)	0.29 (0.03)	0.29 (0.03)	0.19 (0.05)
Eat	0.24 (0.25)	0.21 (0.02)	0.13 (0.03)	0.19 (0.02)	0.19 (0.02)	0.15 (0.03)
Food carry	0.63 (0.07)	0.62 (0.07)	0.41 (0.09)	0.72 (0.08)	0.62 (0.07)	0.37 (0.11)
Nest building	0.58 (0.09)	0.60 (0.09)	0.34 (0.10)	0.38 (0.08)	0.41 (0.09)	0.20 (0.13)
Work	0.38 (0.04)	0.38 (0.04)	0.36 (0.04)	0.40 (0.04)	0.40 (0.04)	0.32 (0.06)
**Scan level**						
Active nonhelping			0.56 (0.02)			0.53 (0.02)
Eat			0.34 (0.02)			0.27 (0.02)
Food carry			0.98 (0.07)			0.91 (0.06)
Nest building			1.23 (0.09)			0.85 (0.10)
Work			0.75 (0.03)			0.69 (0.03)
**Group level**						
Active nonhelping			0.11 (0.06)			0.11 (0.06)
Eat			0.07 (0.04)			0.10 (0.05)
Food carry			0.26 (0.15)			0.15 (0.12)
Nest building			0.17 (0.12)			0.22 (0.16)
Work			0.08 (0.06)			0.14 (0.09)
**Litter level**						
Active nonhelping			0.05 (0.04)			0.12 (0.07)
Eat			0.10 (0.04)			0.08 (0.05)
Food carry			0.19 (0.12)			0.48 (0.14)
Nest building			0.13 (0.10)			0.33 (0.16)
Work			0.06 (0.05)			0.18 (0.10)

Estimates represent the SDs of the random effects (values in parentheses are the SDs of these estimates in the posterior distributions).

**Table A7:** Posterior means of fixed effects in Model 3 for each sex (SD in parentheses)

Model	Active nonhelping	Eat	Food carry	Nest building	Work
3, Females					
Age	−0.06 (0.04)	**−0.17 (0.03)**	0.08 (0.09)	**−0.28 (0.14)**	0.06 (0.05)
Age^2^	**−0.15 (0.02)**	0.00 (0.02)	**−0.24 (0.07)**	**−0.44 (0.10)**	**−0.40 (0.30)**
Age^3^	**0.03 (0.01)**	0.01 (0.03)	0.04 (0.03)	0.10 (0.06)	**0.09 (0.02)**
Group size	**0.13 (0.04)**	0.09 (0.02)	−0.00 (0.09)	−0.15 (0.11)	0.10 (0.06)
Group size^2^	−0.03 (0.03)	0.01 (0.02)	−0.04 (0.05)	**0.18 (0.06)**	−0.01 (0.03)
Relative mass	**0.06 (0.02)**	**0.10 (0.02)**	**0.19 (0.06)**	−0.06 (0.08)	0.04 (0.03)
Pups present (Y)	−0.07 (0.08)	−0.03 (0.06)	−0.02 (0.18)	0.10 (0.25)	0.01 (0.11)

3, Males					
Age	0.01 (0.03)	**−0.15 (0.03)**	0.16 (0.09)	−0.20 (0.13)	**0.16 (0.05)**
Age^2^	**−0.08 (0.02)**	0.01 (0.02)	**−0.20 (0.08)**	**−0.28 (0.10)**	**−0.30 (0.03)**
Age^3^	**0.02 (0.01)**	−0.01 (0.01)	0.04 (0.03)	**0.09 (0.05)**	**0.09 (0.01)**
Group size	0.05 (0.05)	−0.01 (0.03)	**−0.25 (0.09)**	−0.12 (0.11)	−0.03 (0.06)
Group size^2^	0.03 (0.03)	**0.05 (0.02)**	0.02 (0.06)	**0.18 (0.07)**	0.06 (0.04)
Relative mass	**0.09 (0.02)**	**0.04 (0.02)**	**0.17 (0.07)**	0.03 (0.08)	**0.09 (0.03)**
Pups present (Y)	−0.18 (0.09)	**−0.15 (0.06)**	−0.08 (0.19)	−0.17 (0.24)	0.03 (0.12)

Parameters in bold indicate estimates where the 95% credible intervals do not span zero.

## Appendix

Being multinomial, discrete behavioural categories follow a categorical (generalized Bernoulli) distribution, where the probability of observing each behaviour *k* is separately specified as π_*k*_. Of the total *K* behaviours, one serves as a reference category against which the odds of the other *K* − 1 behaviours are contrasted in *K* − 1 ‘subequations’.

The multinomial model can be readily extended to include random effects and thereby allow the probability of exhibiting behaviour *k* to vary across clusters within the data, such as across individuals and groups, or across temporally autocorrelated sampling periods. Consider a simple scenario in which three behaviours (*k* = 1,2,3) are modelled as varying across a population of individuals as a function of age. If behaviours recorded at time *t* are temporally independent, and *k* = ‘3’ serves as the reference category, then the log-odds of individual *i* displaying behaviour ‘1’ or ‘2’ instead of the reference behaviour ‘3’ is given by:

$$\log\left(\frac{{\text{π}}_{1it}}{{\text{π}}_{3it}}\right)={\;\text{β}}_{1it}+{\text{β}}_{\text{a}1}+{\text{γ}}_{1i}$$

$$\log\left(\frac{{\text{π}}_{2it}}{{\text{π}}_{3it}}\right)={\;\text{β}}_{2it}+{\text{β}}_{\text{a}2}+{\;\text{γ}}_{2i}$$

$$\left\lfloor \frac{{\text{γ}}_{1i}}{{\text{γ}}_{2i}}\right\rfloor ∼\text{Normal}\left(0,{Ω}_{\text{γ}}\right):{Ω}_{\text{γ}}=\left[\begin{array}{cc} {\text{σ}}_{{\text{γ}}_{1}}^{2} & \\ {\text{σ}}_{{\text{γ}}_{1\text{,}\;2}} & {\text{σ}}_{{\text{γ}}_{2}}^{2} \end{array}\right]$$

$${\text{π}}_{1}+{\text{π}}_{2}+{\;\text{π}}_{3}=1$$

where β_1*it*_ and β_2*it*_ are the intercepts that contrast the first two behaviours against the reference category, β_*a1*_ and β_*a2*_ are fixed effects for age at the level of each of the first two behaviours relative to the reference category, and γ_1*i*_ and γ_2*i*_ are the individual level random effects, taken to be multivariate normal distributed with zero means. When individual level intercepts are positive, γ_k*i*_ > 0, an individual is more likely than average to exhibit behaviour *K* instead of the reference category, and vice versa for negative individual level intercepts. The variance - covariance matrix of the individual level random effects also facilitates the estimation of within-individual correlations across the *K* − 1 behavioural categories: ρ_1,2_ = σ_γ1,2_/(σ_γ1_.σ_γ2_). Positive correlations indicate that individuals that do more of the first behaviour also do more of the second behaviour (each relative to the reference), while negative correlations indicate the opposite. Through these random effects correlations, MMBMs explicitly enable the estimation of within-individual trade-offs in behavioural time budgets.
